# Preoperative Evaluation of Coronary Artery Disease in Liver Transplant Candidates: Many Unanswered Questions in Clinical Practice

**DOI:** 10.3390/diagnostics11010075

**Published:** 2021-01-05

**Authors:** Maria Bonou, Sophie Mavrogeni, Chris J. Kapelios, Marina Skouloudi, Constantina Aggeli, Evangelos Cholongitas, George Papatheodoridis, John Barbetseas

**Affiliations:** 1 Department of Cardiology, Laiko General Hospital, 11527 Athens, Greece; bonou.maria@yahoo.com (M.B.); marinoula20@hotmail.com (M.S.); jbnv@otenet.gr (J.B.); 2 Department of Cardiology, Onassis Cardiac Surgery Center, 17674 Athens, Greece; sophie.mavrogeni@gmail.com; 3 First Department of Cardiology, Hippokration General Hospital, Medical School of National & Kapodistrian University, 11527 Athens, Greece; dina.aggeli@gmail.com; 4 First Department of Internal Medicine, Medical School of National & Kapodistrian University, 11527 Athens, Greece; cholongitas@yahoo.gr; 5 Department of Gastroenterology, Laiko General Hospital, Medical School of National & Kapodistrian University, 11527 Athens, Greece; gepapath@med.uoa.gr

**Keywords:** liver transplantation, cardiovascular risk, risk stratification, prognosis, screening, coronary artery disease, imaging

## Abstract

Cardiovascular (CV) complications represent the first non-graft-related cause of death and the third overall cause of death among patients undergoing liver transplantation (LT). History of coronary artery disease is related to increased CV mortality following LT. Although it is of paramount importance to stratify CV risk in pre-LT patients, there is no consensus regarding the choice of the optimal non-invasive cardiac imaging test. Algorithms proposed by scientific associations include non-traditional risk factors, which are associated with increased cardiac risk profiles. Thus, an individualized pre-LT evaluation protocol should be followed. As the average age of patients undergoing LT and the number of candidates continue to rise, the “3 W” questions still remain unanswered, Who, Which and When? Who should be screened for coronary artery disease (CAD), which screening modality should be used and when should the asymptomatic waitlisted patients repeat cardiac evaluation? Prospective studies with large sample sizes are warranted to define an algorithm that can provide better risk stratification and more reliable survival prediction.

## 1. Introduction

Liver transplantation (LT) is the second most commonly performed solid organ transplant procedure worldwide, after kidney transplantation [1]. Cardiovascular (CV) complications represent the first non-graft-related cause of death and the third overall cause of death among patients undergoing orthotopic liver transplantation (OLT), with infection and rejection being more common [2]. The spectrum of CV events varies from coronary artery syndromes, pulmonary edema due to heat failure or cardiomyopathy, right heart failure as a consequence of portopulmonary hypertension, dysrhythmias, stroke and pulmonary embolism, with the risk being higher in patients with known coronary artery disease (CAD) [3,4]. History of CAD or presence of traditional risk factors are related with increased CV mortality perioperatively (2.3%) and within five years post OLT (16–22%). In end-stage liver disease (ESLD) there is peripheral vasodilation, high cardiac output and increased flow in both the pulmonary and systemic circulations [5]. Thus, CAD may be masked by reduced afterload. Perioperative hemodynamic stress, hypercoagulation, as well as an increase in systemic vascular resistance in association with reduction in venous return and cardiac output postoperatively, are the main reasons for adverse cardiac outcomes among ESLD patients with known CAD [3]. The majority of post-LT acute coronary syndromes seem to occur early, during the first month post-LT, and to be related to pre-existing CAD [6]. The prevalence of CAD in ESLD has been reported to be 2.5–27%, with the wide range most likely attributed to the heterogeneity of published data and the definition of CAD [3,7]. This prevalence is equal to or higher than that estimated for the general population and is more prevalent in patients with nonalcoholic fatty liver disease (NAFLD) [8]. Moreover, the older age of the current OLT recipients, the higher incidence of age-related comorbidities and traditional risk factors of CAD, and the improvements in critical care support over the last decades pose contemporary LT candidates at even greater post-transplant CV risk.

Although it is of paramount importance to stratify CV risk in pre-transplant ESDL patients, there is no consensus regarding the choice of the optimal non-invasive cardiac imaging test [3,4,9,10]. This review focuses on the diagnostic imaging approaches in the evaluation of silent CAD before LT and their efficacy to predict perioperative risk based on current available evidence, as well as on important unanswered questions.

## 2. Dobutamine Stress Echocardiography (DSE)

Although DSE has been widely used for identification of CAD and risk stratification before noncardiac surgery, its role in patients with ESLD who are scheduled to undergo OLT is uncertain [11]. The American Association for the Study of Liver Diseases (AASLD)/American Society of Transplantation (AST) 2013 guidelines suggest stress echocardiography as an initial screening test for all LT candidates [4]. Although earlier small sample studies had demonstrated high sensitivity of DSE to detect subclinical CAD and predict outcomes after OLT, more recent studies did not support these findings [12,13]. In a meta-analysis of four perioperative studies, Nguyen et al. reported that DSE had a sensitivity of 32% and limited accuracy for the detection of CAD among patients with ESLD awaiting liver transplant [14]. They also found that the test carried a high negative, but low positive predictive value in predicting outcomes perioperatively and long-term. Similarly, a retrospective study from the Mayo Clinic confirmed the low sensitivity of DSE (42%) to identify CAD compared with coronary angiography (CA) in 66 LT candidates [15].

The high negative predictive value (NPV) of DSE in predicting short- and long-term CV outcomes post-LT has also been reported [14,16,17]. Nicolau-Raducu et al. reported high NPV (89%) and low positive predictive value (PPV, 33%) of DSE for prediction of early cardiac events in 195 patients who achieved the target heart rate on stress test [6]. Although the study suggested that DSE appeared to be effective in ruling out adverse cardiac events, it is notable that the majority of cardiac events (21/23) occurred in patients with normal findings on DSE. On the other hand, Patel et al. in a large retrospective study of 460 LT candidates found no association between DSE and 30-day CV events [18]. These findings were consistent with prior studies that found no correlation of DSE’s positivity with intraoperative cardiac events in cirrhotic patients who undergo OLT [19,20].

Additional insights have emerged from a very recent retrospective, single center study in a large cohort of 633 ESLD patients who underwent CA and DSE over a 20-year period [21]. The overall sensitivity of stress test to detect CAD was low (24%). However, when tardokinesis and lack of hyperkinesis were considered as abnormal findings, DSE sensitivity rose to 67%. Importantly, abnormal DSE was positively associated with poorer outcomes. LT-recipients with CAD and abnormal DSE were 2.5 times more likely to experience CV events compared with those with CAD and normal DSE.

To conclude, the existing data support that DSE has limited diagnostic accuracy to detect CAD compared with the gold standard of CA and provides inadequate preoperative risk stratification of patients prior to OLT. Several reasons could explain the reduced sensitivity of DSE in LT-candidates [22]. Due to splanchnic and systemic vasodilatation, ESLD patients typically have decreased peripheral systemic resistances and hypercontractile left ventricle (LV), where the detection of small ischemic regions is difficult, owing to the tethering by adjacent segments which become hyperkinetic with stress. Furthermore, it is known that DSE has limited accuracy, exhibiting mostly high numbers of false negative results, in the setting of small LV chamber size and reduced wall stress in general population [23]. Similarly, in ESLD patients decreases in central venous pressure, preload, systemic vascular resistance and blood pressure caused by dobutamine, lead to reductions in LV dimension/volume and in wall stress inducing less ischemia due to lower myocardial oxygen consumption. Moreover, the wide use of b-blockers as prophylaxis against esophageal variceal bleeding in such patients frequently results in lower heart rates and inadequate rate-pressure products to elicit ischemia in many cases. In addition, the presence of ascites may limit the acquisition of high-quality images and distort the contour of the LV resulting in pseudodyskinesis of the posterior wall. Finally, a positive stress in ESLD patients without significant coronary stenosis may be the result of microvascular dysfunction, which is more prevalent in patients with NAFLD [24,25]. Incorporation of quantitative methods and newer echocardiographic indices, like LV global longitudinal strain and contrast echo, during DSE might enhance the sensitivity of the test in future studies.

PPV, NPV and the disadvantages of all screening tests to predict CAD are summarized in Table 1.

## 3. Myocardial Perfusion Imaging (MPI)

Single photon emission computed tomography (SPECT) MPI is a widespread imaging modality for pre-operative cardiac risk stratification with the majority of studies including patients awaiting vascular surgery [32]. The evaluation of CAD in LT candidates by SPECT MPI has yielded conflicting results, with some early small studies reporting high diagnostic performance when compared with CA and some recent studies not confirming such a high accuracy [26,27,28]. Aydinalp et al. defining a positive SPECT study as the presence of only reversible perfusion defects, reported a sensitivity of 100% but an accuracy of 38% for SPECT to detect presence of CAD [26]. On the contrary, Davidson et al. found lower SPECT sensitivity (37%) and specificity of 63% [27]. In keeping with these findings, Bhutani et al. found that adenosine and regadenoson SPECT imaging had low sensitivities (62% and 35%, respectively), but high NPVs for diagnosing severe CAD (95% and 93%, respectively) [28]. The authors concluded that SPECT imaging was an inaccurate screening test, with its diagnostic ability to detect significant CAD equivalent to that of traditional CV risk factors. Nevertheless, Baker et al. demonstrated that when applied only to the subset of patients categorized as high CAD risk, the modality was more effective, with PPV 67% and NPV 97% [29].

Prognostic value of SPECT MPI has also been questioned. Bradley et al. reported a low proportion (7%) of positive SPECT studies among 710 ESLD patients, with no correlation between abnormal findings of the imaging test and early cardiac events after OLT [33]. Similarly, a more recent two-center study conducted in the Mayo Clinic found low rate of abnormal results using technetium-99m-SPECT (8%) and a significantly lower than previously reported CV event rate after OLT (in <10% peri-procedural and in <2% during the 6-month postoperative period) [34]. Nonetheless, a trend towards higher rates of peri-operative ischemic complications was noted in patients with a positive SPECT study. These findings were also confirmed in another study by Duvall et al. [35].

In contrast, Zoghbi et al. demonstrated that a normal SPECT study had a 99% NPV for perioperative cardiac events and could identify patients at a very low risk for early and late cardiac events after OLT [36]. Accordingly, a very recent, large observational, 5-year follow up study, the Exercise Capacity and Single Photon Emission Computed Tomography in Liver Transplantation Candidates (ExSPECT), reported that abnormal SPECT perfusion was an independent predictor of intermediate and long-term CV morbidity and mortality in 404 high-risk ESLD patients who underwent OLT, also providing incremental prognostic value and improved classification [37].

In two studies comparing stress echo with nuclear tests, SPECT MPI did not have an advantage over DSE with both modalities having similar clinical results [34,38]. In a study by Snipelisky et al., both stress modalities had equal efficacy to predict clinical outcomes in patients undergoing OLT [34]. Similarly, in a very recent meta-analysis including heterogeneous retrospective studies, Soldera et al. using CA as gold standard, reported that DSE and MPI had limited accuracy in both predicting post-LT adverse outcome and detecting CAD with a pooled sensitivity of 28% and 61% and specificity of 82% and 74%, respectively [38].

In summary, despite the high heterogeneity between studies over the past two decades, SPECT MPI yields low diagnostic efficacy in detecting CAD among LT candidates. However, when it is used for the subset of patients at high risk for CAD, it seems to provide prognostic information regarding adverse outcomes. Several factors may contribute to such mixed findings. Although SPECT MPI is not affected by b-blocker therapy, the impaired vasodilatory reserve in ESLD patients may reduce the effectiveness of a vasodilator stress test, such as SPECT MPI using dipyridamole, adenosine, or regadenoson, resulting in both false-positive and false-negative findings [39]. Furthermore, a false positive result may be due to image artifacts secondary to splenomegaly and ascites in LT candidates. Innovation in stress imaging protocols, camera technology, especially with the use of cadmium zinc telluride (CZT) detectors, and processing iterative reconstruction software can enhance SPECT’s diagnostic accuracy, as well as reduce radiation exposure [40]. Finally, the use of PET may provide a better assessment of CAD in ESLD patients, since it provides higher image resolution and diagnostic accuracy for detecting CAD in the general population compared to SPECT MPI, while additionally allowing quantitative myocardial blood flow and myocardial flow reserve measurements [41].

## 4. Cardiac Computed Tomography (CT)

Cardiac CT can noninvasively identify subclinical CAD by (i) calculating the amount of coronary artery calcium (CAC) using non-contrast CT, and (ii) evaluating the degree of stenosis, the composition of the atherosclerotic plaque and measuring the fractional flow reserve (FFR-CT) with coronary CT angiography (CCTA) [42].

### 4.1. Coronary Artery Calcium Score (CACs)

The usefulness of CACs to identify CAD and stratify patients at risk for future CV events has been documented in the general population [43]. In a study conducted by McAvoy et al., CACs was significantly associated with CV risk factors such as age, systolic blood pressure and diabetes mellitus, as well as with the number of diseased coronary vessels in LT recipients, highlighting the incremental value of CACs over Framingham risk score [44]. Accordingly, Kong et al. reported that increasing age, male sex, and diabetes mellitus were associated with a CACs > 400 in 548 LT recipients [45]. In keeping with these findings, Kemmer et al. found that the likelihood of angiographically proven CAD increased with higher CACs in ESLD patients [46]. Significant CAD requiring revascularization was present in 24% of the candidates with CACs > 400 and in 0% of those with CACs 100–400. Subsequently, Kong et al. demonstrated that a preoperative CACs of >400 was an important predictor of CV complications 1-month post-LT among 443 LT recipients, suggesting that it represents a reliable screening tool for preoperative cardiovascular evaluation in these patients [47]. Based on the aforementioned studies, Choi et al. suggested using CCTA combined with CACs for preoperative cardiac evaluation in ESLD patients with diagnosis of diabetes mellitus or ≥2 traditional risk factors for CAD. On the other hand, invasive CA ought to be performed in patients with coronary artery stenosis ≥50% on CCTA or CACs > 400, since in this case, the incidence of significant CAD requiring revascularization is high [10,48].

More recently, CACs derived from non-ECG-gated CT was a reliable marker to rule out CAD in 953 patients being evaluated for OLT [49]. Cut-off values < 4 for Agatston score and <2 for Weston score were predictive of non-obstructive CAD with 100% certainty and could help avoid invasive CA in this high-risk population. Furthermore, Buggs et al. using CA as the gold standard, found that CACs was a better screening test method of CAD risk assessment than DSE, having a much higher PPV (80% versus 56%, respectively) [50]. On the contrary, in an ExSPECT trial there was no association between CACs with either the extent of SPECT perfusion abnormality or with outcomes [37]. Further data is required to clarify the additive predictive value of CACs and to inform about preoperative strategies in evaluation of LT candidates.

### 4.2. CCTA

CCTA has a high NPV (up to 99%) in excluding cardiac events among patients undergoing non-cardiac surgery or in the setting of non-surgical patients, but the data regarding its use in LT candidates are limited [30,51]. Currently, there is a paucity of data comparing CCTA and invasive CA for detecting CAD in this setting. The prevalence of obstructive CAD (>50% stenosis) detected by CCTA in LT candidates was initially reported to be high (34%) in a small single center study [52]. However, these findings were not confirmed in another retrospective registry study, in which a low prevalence (7%) of obstructive CAD (≥50% stenosis) was detected in 1045 LT candidates without any history of chest pain or CAD, being similar with that in matched controls with healthy livers [53]. Moreover, non-obstructive CAD and multi-vessel atherosclerosis were more frequent in cirrhotic patients [53]. Obstructive CAD was correlated with traditional CV risk factors in LT candidates. These results suggested the usefulness of CCTA in noninvasively ruling out significant CAD, thereby avoiding unnecessary invasive CA.

Regarding the prognostic value, Jodocy et al. found that CCTA was a reliable tool to predict peri- and postoperative CV events in 54 patients undergoing OLT [54]. Accordingly, Cassagneau et al. demonstrated that preoperative CCTA had high NPV of 91%, similar to that of DSE, for postoperative major cardiac events in 52 LT recipients, of whom 71% had normal coronary arteries or non-obstructive coronary plaques [55]. Subsequently, in a retrospective study including 2118 patients, Moon et al. reported that a negative CCTA could successfully exclude post-LT myocardial infarction and thus proceeding with further study, such as invasive CA, was unnecessary [56]. Additionally, in a recent study, when CCTA findings were correlated with SPECT MPI, nearly all ESLD patients were found having a normal SPECT MPI result. This finding indicates that CCTA may serve as a more effective gatekeeper to improve efficiency of referral to CA [57]. The authors also reported high eligibility for FFR-CT analysis due to acceptable CT image quality in these patients with impaired hemodynamic profiles, while this issue is also being addressed by the ongoing CRASCH-Liver trial (www.clinicaltrials.gov Identifier: NCT04089969).

The performance of CCTA may be limited by the presence of tachycardia, prevalent diffuse coronary calcifications, in the presence of which PPV is low (25%), concomitant renal impairment and presence of ascites that makes it difficult for ESLD patients with cirrhosis to lie down [10,58].

In conclusion, given the overall high NPV (>95%) of CCTA for excluding significant CAD in ESLD patients, non-invasive CA may be considered as an acceptable alternative to invasive CA, according to the 2018 consensus by the American Society of Transplantation (AST), Liver and Intestinal (LICOP), and Thoracic and Critical Care (TCC COP) Communities of Practice (Table 2) [10].

## 5. Cardiovascular Magnetic Resonance (CMR)

CMR is an operator independent modality with high reproducibility [59]. CMR can provide a “one-stop-shop” approach to evaluate cardiac structure, function, and ischemia in LT candidates [60,61]. It can detect ischemia using two different ways: (a) by observing wall motion abnormalities, using the stress factor dobutamine, exhibiting better sensitivity and specificity than DSE and (b) by observing myocardial perfusion using the first pass of a bolus of a T1-shortening contrast agent (first-pass gadolinium) injected into a peripheral vein [62,63]. The spatial resolution of CMR myocardial perfusion (using most commonly adenosine) is superior to other imaging modalities, such as nuclear techniques, so that subendocardial ischemia can be more reliably identified [31,63]. Myocardial perfusion abnormalities can be attributed either to epicardial CAD or to microcirculation disorders, which are frequently present in LT candidates [64]. If CMR stress examination does not show ischemia in LT candidates, the 12-month event free survival rate is almost 100% [65]. In parallel with ischemia assessment, CMR can also detect myocardial scar due to either myocardial infarction or cirrhotic cardiomyopathy. Finally, the assessment of cardiac iron and extracellular volume fraction by CMR were predictive of heart failure and worse transplant-free survival, respectively [66,67].

## 6. Invasive CA and Revascularization

Invasive CA remains the gold standard for diagnosis of CAD in pre-LT patients [10]. The prevalence of CAD in asymptomatic LT candidates varies from 13 to 26% and is related to traditional risk factors [68]. CA can be performed safely in LT candidates, despite the alterations in hemostasis and renal function, with the transradial approach being the preferred method to reduce bleeding complications [10,69,70]. ESLD patients are more prone to CA-related complications due to thrombocytopenia, anemia, coagulopathy and kidney disease, with bleeding and the need for blood transfusions representing the most common complications [71]. Furthermore, ultrasound-guided femoral approach has been proven to be safe with low risk of complications as reported in a recent study including 559 LT patients [71]. In keeping with these results, Singh et al. demonstrated that percutaneous coronary intervention was safe in ESLD patients, even if it was performed on an emergent basis in 77% of them [72].

The presence and extent of CAD have been shown to be strong predictors of adverse CV outcomes after OLT [73,74]. Yong et al. demonstrated that the presence of multi-vessel disease, even in the absence of severe coronary artery stenosis, was associated with higher mortality after OLT, underlining that the invasive assessment of CAD may have prognostic value [73]. However, in a meta-analysis, Soldera et al. reported that CA did not satisfactorily predict the risk of perioperative cardiac events or all-cause mortality among LT candidates, but with referral bias most likely having affected the results [38].

Although, to date, there is lack of evidence for therapeutic efficacy of coronary revascularization in asymptomatic patients [75], a few studies have documented the impact of pre-transplant coronary invasive approaches on post-LT outcome. Despite the pre-transplant implementation of angioplasty or coronary artery bypass surgery (CABG), Snipelisky et al. found that mortality rates remained high after OLT in patients with severe CAD before LT, suggesting that coronary interventions before OLT do not necessarily offer a CV survival benefit [76].

On the contrary, recent studies highlighted that revascularization of obstructive CAD prior to LT may improve CV mortality. In a multicenter retrospective study during a 12-year period, 3-year survival of LT patients with significant CAD (>50% stenosis), of whom 53% were revascularized, was not inferior to that of patients without CAD [77]. Importantly, there was a trend toward decreased survival among the 80/630 patients who underwent either preoperative percutaneous coronary intervention (PCI), CABG, or both, although the study was not powered for this comparison, underscoring the role of risk factors on long-term survival. Kutkut et al., building on prior work of their group, reported that increased use of CA and PCI was associated with lower cardiac mortality and post-operative myocardial infarctions in 811 LT patients over a 7-year period [71,78]. Similarly, in the study of Wray et al. [77], patients who underwent CA before LT, compared with those who did not, had lower 7-year survival, regardless of the CA result, showing a negative effect of CV risk factors on survival. Given that the sensitivity of DSE in detecting significant CAD was 37%, the authors suggested that invasive CA may be performed as a primary screening tool in selected patients even in the absence of a positive stress test finding. Thus, the 2018 consensus document by the AST/LICOP/TCC COP (Table 2) recommends that CA should be performed in ESLD patients who have undergone bypass surgery and present an ischemic response in a non-invasive test (Grade IC) [10]. Further research is needed to indicate when to proceed with CA and investigate which treatment strategies prior to LT will improve post-transplant survival.

## 7. Guidelines, Current Gaps and Future Perspectives

There is little consensus on the most effective screening methods in LT candidates among guidelines (Table 2) [3,4,8,9]. Given that the number of traditional risk factors is correlated with angiographically detected CAD and linearly with worse survival, American and European guidelines recommend non-invasive testing for asymptomatic LT candidates with >2 cardiac risk factors, although no preferred modality is specified [3,10]. The 2013 AASLD/AST practice guidelines suggest stress echocardiography as the initial screening test with CA when clinically indicated [4]. Furthermore, the more recent American Society of Transplantation consensus recommendations step forward stating that a non-invasive imaging test may be considered according to the pre-test probability, meaning that the presence of >2 risk factors or diabetes mellitus as a single factor are able to justify the test, while the choice of imaging is left to local expertise [10]. Absence of cardiac risk factors rules out angiographically significant CAD among alcoholic and non-alcoholic ESLD-patients, having NPV of ≥97% [79].

However, many gaps still remain, since none of the above algorithms include non-traditional risk factors, such as chronic kidney disease, non-alcoholic steatohepatitis, alcoholic cirrhosis, familial amyloid polyneuropathy, and hereditary hemochromatosis, which are associated with increased cardiac risk profiles [80,81]. Thus, even before choosing the correct diagnostic method, a correct evaluation of the cardiovascular risk of the individual patient is mandatory in order to be able to identify the most suitable test. Moreover, the optimal interval of repeating cardiac evaluation for waitlisted patients also remains unclear. While this issue is currently addressed by an ongoing trial (www.clinicaltrials.gov Identifier: NCT03674307), Akincioglu et al. recommend that repeat testing be performed in patients with an initial abnormal test following medical therapy or undergoing revascularization, or those who will develop new symptoms while awaiting LT [40].

Given that the absence of clinical symptoms is less predictive in LT candidates than in the general population, the role of imaging tests becomes even more important [3]. LT candidates are less likely to report typical symptoms because they have markedly impaired aerobic capacity, rendering them incapable to perform activities and to develop symptoms, while the presence of severe vasodilation can mask the clinical manifestations of CAD [82]. Nevertheless, non-invasive imaging tests in LT candidates have suboptimal sensitivity and specificity for detecting CAD and predictive value for post-transplant cardiac events, based on observational studies (Table 1). Several factors might affect the accuracy of the screening tests. Myocardial injury post-LT may occur by multi-factorial causes such as coronary spasm, coronary thrombosis due to a hypercoagulable state which is frequent in this group of patients, an imbalance between oxygen supply and demand or plaque rupture of a lesion that is not the most severe stenosis [55,83]. Furthermore, varying definitions of the term adverse cardiac outcomes result in substantially different findings and conclusions, while the type of surgery and immunosuppression may also influence cardiac events post-LT [84,85,86].

Nowadays, as the average age of patients undergoing OLT and the number of candidates continue to rise, the “3 W” questions still remain unanswered, Who, Which and When? Who should be screened for CAD, which screening modality should be used and when should the asymptomatic waitlisted patients repeat cardiac evaluation? Prospective studies with large sample sizes are warranted to define an algorithm that can provide better risk stratification and more reliable survival prediction.

## Figures and Tables

**Table 1:** Preoperative assessment of CAD in ESLD patients.

Screening Tests		PPV *	NPV *	Disadvantages in ESLD Patients
**Noninvasive** **tests**	DSE ^1^	0–40%	48–100%	Limited accuracy of DSE to detect CAD due to: - ESLD patients typically have hypercontractile LV - the use of b-blockers results in lower heart rates during the test - the presence of ascites may result in pseudodyskinesis of the posterior wall - microcirculatory disorders
	MPI’s ^2^	15–28%	77–100%	Limited accuracy of MPI to detect CAD due to: - the impaired vasodilatory reserve in ESLD patients may reduce the effectiveness of a vasodilator stress test - the presence of image artifacts secondary to splenomegaly and ascites
	CCTA ^3^	86% in general population	97% in general population	False-positive results are possible in case of elevated diffuse calcification
				Major limitations: - nephrotoxicity - the need for relative bradycardia
	CACs	no data comparing CCTA to CA in ESDL patients		Contraindications: - severe ascites - orthopnea - hepatic encephalopathy
	CMR stress ^4^	77% in general population	91% in general population	Limitations: - lack of availability/expertise - high cost - concern about contrast use in patients with reduced GFR - impossible to scan non MRI conditional devices (metallic clips, pacemakers and defibrillators)
		no data comparing CMR stress to CA in ESDL patients		Contraindications:- severe ascites- orthopnea- hepatic encephalopathy- claustrophobia
**Invasive tests**	CA	NA	NA	Complications: - bleeding - blood transfusions

* as compared to invasive CA as gold standard. ^1^ Reference numbers: [10,14,15,16]; ^2^ Reference numbers: [10,26,27,28,29]; ^3^ Reference number: [30]; ^4^ Reference number: [31]. Abbreviations: CA, coronary angiography; CACs, coronary artery calcium score; CAD, coronary artery disease; CCTA, computed tomography coronary angiography; CMR, cardiovascular magnetic resonance; CV, cardiovascular; DSE, dobutamine stress echocardiography; ESLD, end-stage liver disease; LV, left ventricle; GFR, glomerular filtration rate; MPI, myocardial perfusion imaging; NA, nonapplicable; NPV, negative predictive value; PPV, positive predictive value.

**Table 2:** Guidelines and recommendations for CAD risk assessment prior to LT.

Scientific Organization Recommendation	Risk Factors	DSE or MRI	CCCTA with/or CACs	Invasive CA
AHA/ACC (2012) Guidelines [1]	Risk factors include: - diabetes mellitus - prior CV disease - LVH - age > 60 years - smoking - hypertension - dyslipidemia	Noninvasive stress testing may be considered in liver transplantation candidates with 3 or more risk factors regardless of functional status. (Class IIb, Level of Evidence C)		Invasive CA: - may be performed despite coagulopathy in patients with ESLD, although at increased risk of bleeding complications
AASLD/AST (2013) Guidelines [4]	Risk factors include: - hyperlipidemia - hypertension - diabetes mellitus - smoking - age > 60 years	Stress echo as an initial screening test with CA as clinically indicated. (Grade 1-B) ^1^		Invasive CA: - if CAD cannot be confidently excluded by stress test
ESC/ESA (2014) Guidelines [9]	Risk factors include: - ischemic heart disease - heart failure - renal dysfunction - diabetes mellitus requiring insulin therapy	Imaging stress testing is recommended before high-risk surgery in patients with >2 clinical risk factors and poor functional capacity (<4 METs). (Class I, Level of Evidence C)		Invasive CA: - indications for pre-operative CA are similar to that proposed in the non-surgical setting
AST/LICOP/TCC COP (2018) Consensus Recommendations [10]	Risk factors include: - age (male > 45 years, female > 55 years) - hypercholesterolemia - hypertension - smoking - family history of early CAD (first-degree relative male < 55 years, female < 65 years)	DSE or Vasodilator testing - should be based on individualized evaluation of the candidate’s pretest probability for having CAD. (1C)	CACs and/or CCTA in pts - with normal body habitus, - who are able to lie still, - perform required breath holding maneuvers, and - with a regular nontachycardic rhythm (2C)	Invasive CA: - in pts with CABG with reduction in systolic function or abnormal noninvasive test (1C) - a transradial approach (if possible) and minimization of sheath size are recommended (1C)
			CCTA may be an acceptable alternative to invasive CA	CA can be performed safely in LT candidates despite coagulopathy and renal dysfunction

^1^ Grade 1-B: strong strength of the recommendations, moderate quality of evidence. Abbreviations: AASLD, American Association for the Study of Liver Diseases; AASLD/AST, American Association for the Study of Liver Diseases/American Society of Transplantation; AHA/ACC, American Heart Association/American College of Cardiology; AST/LICOP/TCC COP, American Society of Transplantation/Liver and Intestinal/Thoracic and Critical Care; bpm, beats per minute; CA, coronary angiography; CABG, coronary artery bypass surgery; CACs, coronary artery calcium score; CAD, coronary artery disease; CCTA, computed tomography coronary angiography; CV, cardiovascular; DSE, dobutamine stress echocardiography; eGFR, estimated glomerular filtration rate; ESC/ESA, European Society of Cardiology/European Society of Anaesthesiology; ESLD, end-stage liver disease; HR, heart rate; LT, liver transplantation; MET, metabolic equivalent; LVH, left ventricular hypertrophy; TIA, transient ischemic attack.

## Data Availability

Not applicable.
